# Family Planning Perceptions Among Residents in Orthopedic Surgery, Other Surgical Specialties, and Non-surgical Specialties: A Nationwide Survey Analysis

**DOI:** 10.7759/cureus.84912

**Published:** 2025-05-27

**Authors:** Janae Rasmussen, Brendan J Liakos, Kelly M Frasier, Noah Paisner, Eric Huish, Shu Lin, McHenry Mauger, Richard Gellman

**Affiliations:** 1 Orthopedic Surgery, Valley Consortium for Medical Education, Modesto, USA; 2 Dermatology, Northwell Health, New Hyde Park, USA; 3 Orthopedic Surgery, San Joaquin General Hospital, French Camp, USA; 4 Medical Education, Nova Southeastern University Dr. Kiran C. Patel College of Allopathic Medicine, Fort Lauderdale, USA; 5 Biostatistics, Nova Southeastern University Dr. Kiran C. Patel College of Allopathic Medicine, Fort Lauderdale, USA

**Keywords:** equality, family planning, medical education and training, orthopaedic surgery, resident well-being

## Abstract

Background

Understanding family planning perceptions among medical residents across different specialties is crucial for addressing the challenges of balancing personal and professional responsibilities. This survey aimed to evaluate how residents in orthopedic surgery (OS), non-orthopedic surgical specialties (SS), and non-surgical specialties (NSS) view the factors that help or hinder family planning and perceptions of their specialty’s support for family planning. We hypothesized that NSS residents would view family planning as more manageable compared to those in OS and SS.

Objective

The goal of this study is to observe factors in orthopedic surgery compared to other surgical and non-surgical specialties that may influence a resident’s family planning decisions during training.

Methods

A self-reported survey was distributed via Instagram and emailed to residents training in the United States of America, and was open to all postgraduate years and specialties. The 28-question survey assessed perceptions of family leave policies, the feasibility of starting a family during training, and factors influencing delays in family planning. Qualitative and quantitative data were collected, and a chi-squared test was used for statistical analysis.

Results

This survey of 202 residents across various specialties revealed that family planning support varies significantly, with surgical specialties perceiving fewer opportunities for having children compared to non-surgical specialties. Female representation amongst faculty positively influenced family planning decisions, with over half of female residents in SS and NSS reporting that it impacted their likelihood of having children during residency, suggesting a positive correlation between female faculty and family planning. Additionally, financial constraints, program culture, and the presence of nearby family were key factors influencing family planning decisions across all groups.

Conclusions

This study highlights significant disparities in perceived support for family planning across medical specialties, with OS and SS viewed as more challenging. The presence of female faculty positively influences female residents' decisions. These findings call for policies and resources to support residents, particularly in male-dominated fields such as orthopedic surgery.

## Introduction

Family planning during postgraduate medical training is an increasingly relevant issue. In the United States, the average age of a mother at first birth is 26.6 years compared to 30.4 years for physician mothers [1]. Given that the average age of postgraduate year (PGY)-1 residents is 30.9 years [2], many residents face the difficult decision of whether to start a family during their training or wait until residency is completed. The challenges of pregnancy and parenting during surgical residency have been extensively documented. While having children has been associated with positive work attitudes, it may also increase work-induced family strain [3]. Male residents are more likely to have children during residency than their female counterparts, suggesting additional challenges faced by women in surgical training [4].

Somewhat surprisingly, female surgical residents have been reported to have children more frequently during training than those in non-surgical specialties (NSS), particularly in programs led by female faculty [5]. Orthopedic surgery (OS) residents face disparities in leave duration and are more likely to delay childbearing, which may have significant implications for training timelines [6]. Moreover, bias from colleagues and faculty has been identified as a significant concern for female residents with children [7], emphasizing the need for supportive and equitable policies. In a 2023 systematic review, they found that female orthopedic surgeons have increased rates of infertility and obstetric complications in addition to delaying childbearing to later years compared to the general population [8]. Inconsistencies in residency program policies on parenting further complicate the decision to have children. Sandler et al. surveyed 66 program directors and found that while 65% of programs had a maternal leave policy, only 35% had a paternal leave policy [9]. Unsurprisingly, the perceived impact of having children on well-being varied significantly between genders.

Overall, while the decision to have children during residency is influenced by various factors, the existing literature underscores the importance of addressing gender disparities and providing consistent support for all residents. This study aims to examine correlations between financial concerns, institutional policies, and gender representation in shaping residents’ perceptions of family planning decisions. This study assesses how financial barriers, program structure, mentorship, gender inclusivity, and institutional leave policies impact family planning among surgical and non-surgical residents. We hypothesize that non-surgical residents perceive family planning as more manageable than residents in orthopedic surgery and other surgical specialties (SS), primarily due to institutional and financial differences.

## Materials and methods

Study design

After obtaining institutional review board (IRB) approval (#23-370) from the San Joaquin General Hospital IRB (French Camp, California, USA), an anonymous online survey was distributed via Instagram by making posts and sharing them publicly, and also by email, which was open to residents of any level of training and of any specialty. The survey was conducted using Google Forms until our minimum sample size was met. The survey included questions pertaining to year of training, specialty, resident awareness of parental leave policies, as well as questions assessing their comfort with disclosing family planning goals to other residents in their program (co-residents) and faculty. 

Participants

The target population included medical residents and fellows across all specialties and locations in the United States. Participants were recruited through professional networks and social media platforms. Inclusion criteria required participants to be currently enrolled in a residency or fellowship program in the United States.

Survey 

The survey consisted of 28 questions, addressing topics such as program type, parental leave policies, perceived support for family planning, and the impact of family planning on career choices. Questions were designed to capture both qualitative and quantitative data, with options to provide additional comments for certain responses.

Data collection

The data was collected through a survey (Appendices) that was given to residents across different residency programs that were grouped as NSS, SS, and OS. Data was collected over a 3-month period from June 2024 to August 2024. Responses were anonymous, and participants provided informed consent before completing the survey. The survey was hosted on Google Forms, ensuring data security and privacy.

Data analysis

Our sample size total was 202 residents from the program categories. We consolidated the data by grouping together answers to reduce variability and enhance the robustness of our statistical analysis. Summary Statistics were calculated for survey questions from residency experiences. 

To determine if there were statistically significant differences in the survey responses between the three categories of programs, we performed a chi-square test. The p-values were calculated to see if the distribution of the responses had a significant difference of proportions (p < 0.05) between programs. We chose a chi-square test due to nominal data. The results of the chi-square tests revealed significant differences between the residency programs for several survey questions.

We conducted a difference of proportions test to see if there is a statistical difference (p < 0.05) between residency programs with survey question answers. Data analysis was performed using JMP Pro 16.0 (JMP Statistical Discovery LLC, Cary, USA)/R 4.2.2 software (R Foundation for Statistical Computing, Vienna, Austria). Results are presented in tables, with statistically significant differences represented by an asterisk (*). Further post-hoc analysis was performed with a Bonferroni correction.

## Results

Demographics

**Figure 1:**
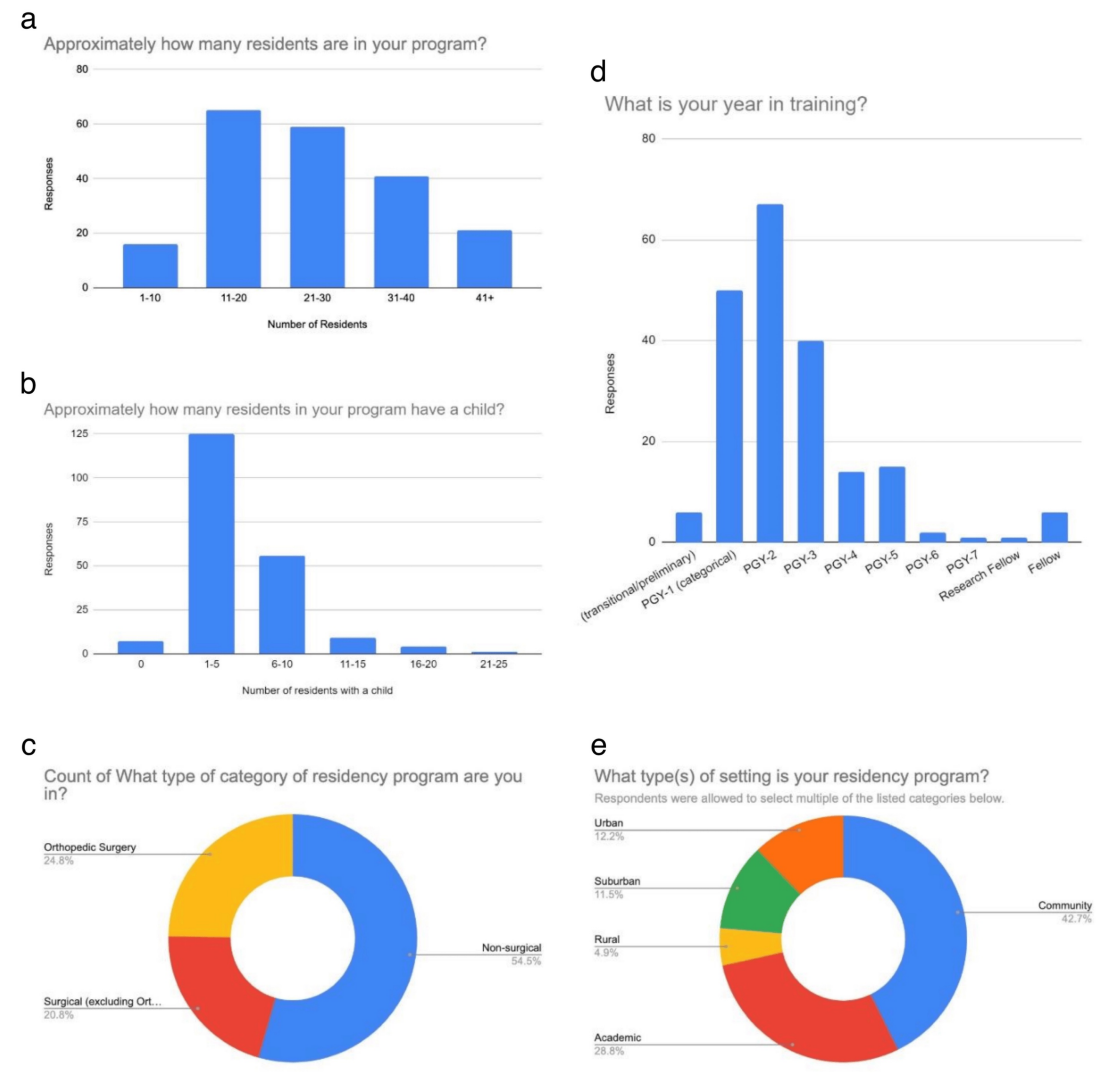
Demographics This figure displays demographic data from the survey respondents. Figure 1a displays the number of residents per program as reported. Figure 1b shows how many residents have a child in their program as reported by respondents. Figure 1c displays what category of residency the respondents were currently enrolled in (surgical, non-surgical, orthopedic). Figure 1d displays what year in training that the respondents report currently being enrolled in. Figure 1e shows what setting the reported programs are in. PGY: postgraduate year

Our survey of 202 residents included all training levels, with the majority being postgraduate year (PGY)-2 67 (33.2%), PGY-1 50 (24.8%), and PGY-3 40(19.8%). Most respondents were from California 68 (33.7%). The majority of residency settings were community-based 123 (60.9%). Among the residents, 110 (54.5%) identified as non-surgical specialties (NSS), with subspecialties including family medicine 44 (21.5%), internal medicine 20 (10%), emergency medicine 14 (7%), and pediatrics 13 (6.5%). Orthopedic surgery (OS) residents made up 50 (24.8%), and surgical specialties (SS) made up 42 (20.8%) of respondents. Distribution across programs was fairly even, with female-identifying residents ranging from one to over 31 per program. 188 (93.1%) of respondents had at least one female faculty member, and about half had one to five co-residents who identified as an underrepresented minority. Additionally, 162 (80.2%) had at least one faculty member from an underrepresented group. 152 (75.1%) reported that a resident or fellow had become pregnant during their training, 34 (16.9%) reported no pregnancies, and 16 (8%) were unsure. 125 (61.9%) reported one to five residents with children, while 56 (27.7%) reported six to 10 residents with children (Figure 1).

Family planning responses

**Figure 2:**
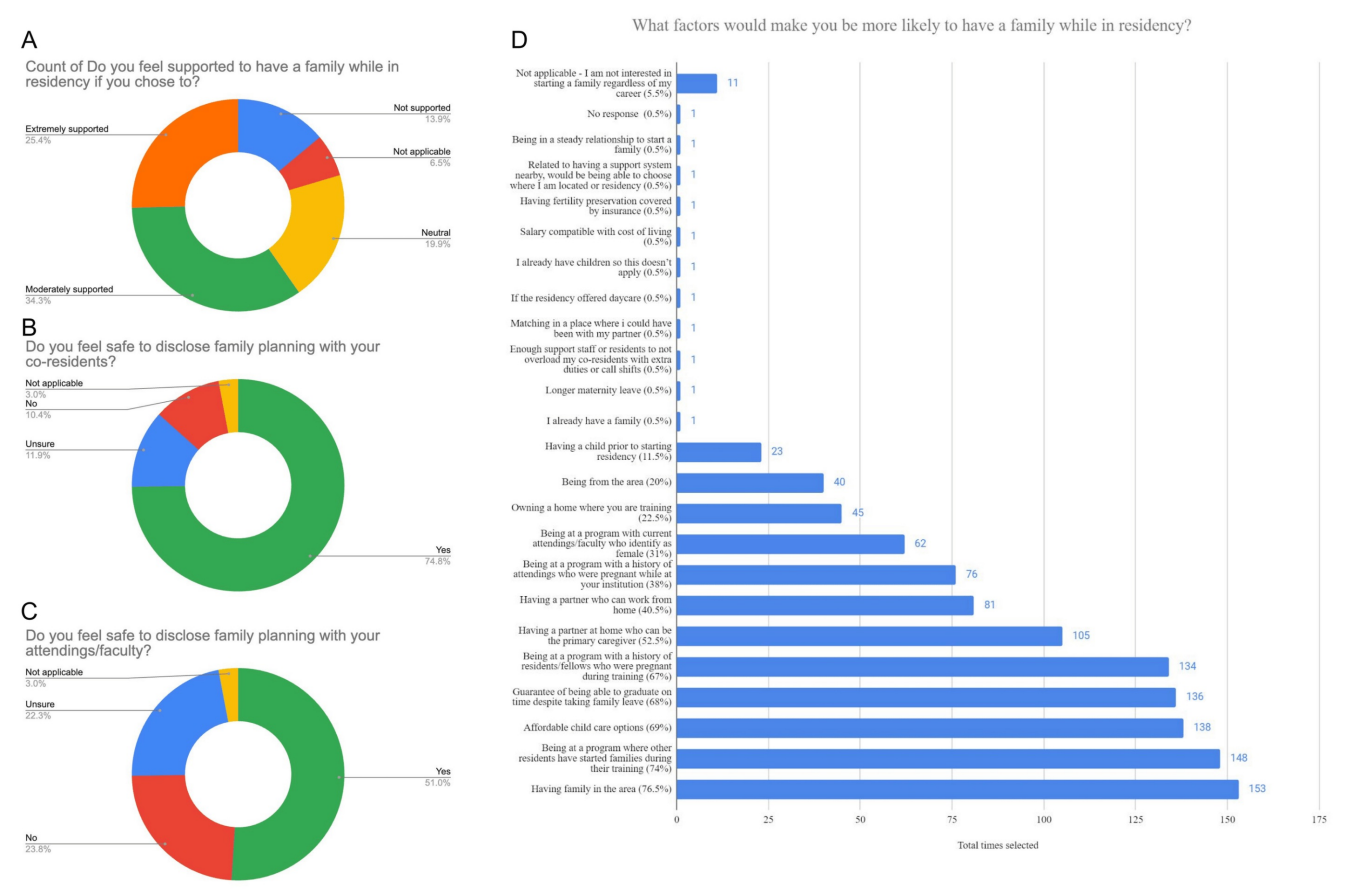
Family Planning Responses Figure 2 shows responses from our survey by all applicants. Figure 2a discusses how residents do or don't feel safe to disclose family planning with their co-residents, while Figure 2b shows respondents' feelings of safety in disclosing family planning with their attendings and faculty. Figure 2c shows the overall feelings of support to have a family while in residency, and Figure 2d displays what factors would make a resident more likely to have a family while in residency.

**Figure 3:**
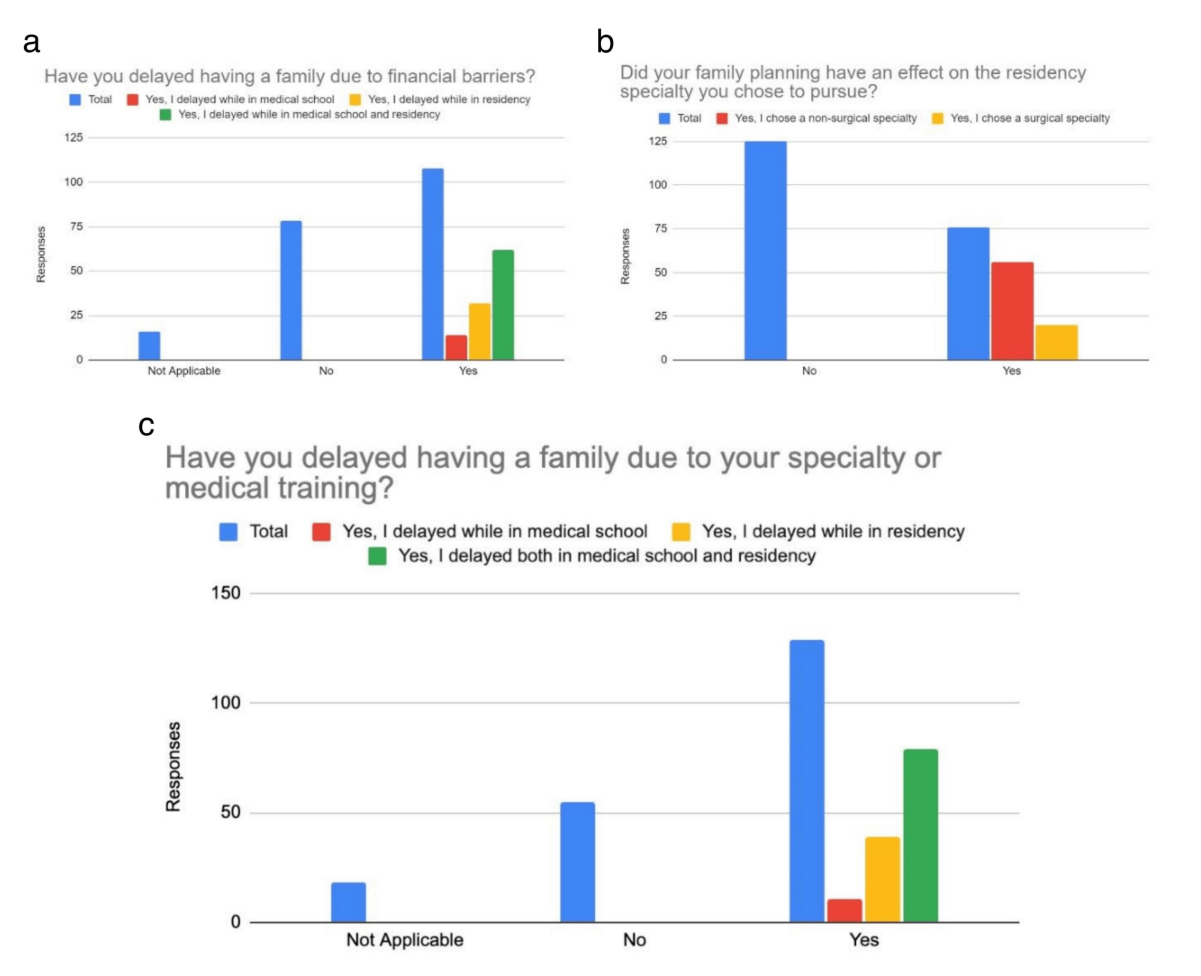
Residency Training Effect on Family Planning Figure 3 discusses whether residents delayed family planning due to financial barriers, as seen in Figure 3a. Figure 3b shows if respondents felt that family planning had an effect on their specialty choice, and Figure 3c shows whether family planning was delayed due to their specialty choice.

A total of 109 (54%) respondents knew their program allowed for 6 weeks of paid time off as required by the Accreditation Council for Graduate Medical Education (ACGME), while 48 (23.8%) were unsure of their leave entitlements. 64 (31.7%) were unaware of the ACGME’s paid leave policy before the survey. Among those who took parental leave, 42 (20.1%) did not feel pressured to return to work, while 16 (8%) did. Support levels for having a family during residency varied: 70 (34.3%) felt "moderately supported," 51 (25.4%) "extremely supported," 40 (19.9%) "neutral," and 28 (13.9%) "not supported." 150 (74%) felt safe disclosing family planning to co-residents, while 21 (10.4%) did not. About 103 (51%) felt safe sharing family planning with faculty, while 48 (23.8%) did not. Financial barriers influenced 62 (30.7%) of respondents to delay starting a family, while 78 (38.6%) reported no financial impact. Specialty choice caused 79 (39.1%) to delay family planning, whereas 55 (27.2%) noted no delay due to specialty. Family planning did not affect specialty choice for 126 (62.2%), while 56 (27.9%) chose a non-surgical specialty for family planning reasons (p < 0.001). Factors influencing family planning decisions included having family nearby 155 (76.5%), seeing peers start families 149 (74%), affordable childcare 139 (69%), assurance of timely graduation with family leave 137 (68%), program history of residents/fellows who were pregnant 135 (67%), and a partner as the primary caregiver 106 (52.5%) (Figures 2,3).

Non-surgical specialty residents' responses

**Figure 4:**
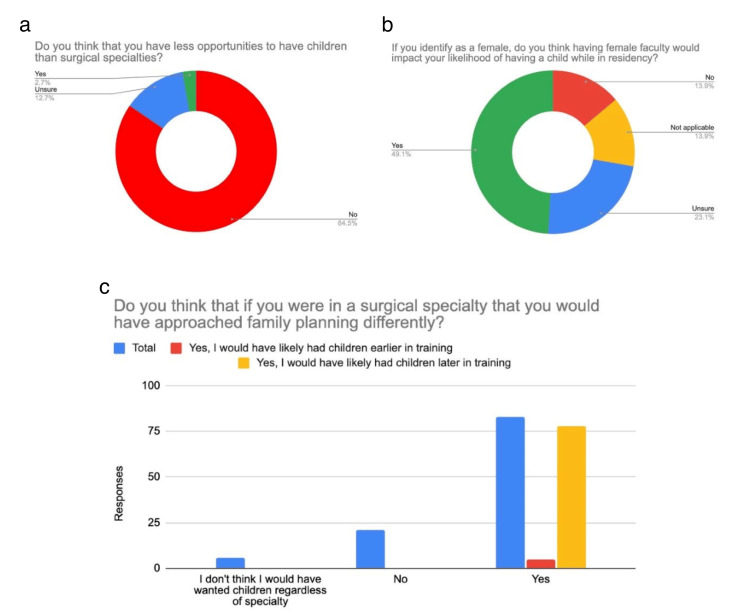
Non-surgical Specialty Residents' Responses Figure 4 displays responses from non-surgical residents. Figure 4a shows resident responses regarding their perceived opportunities for having children compared to their surgical resident counterparts. Figure 4b demonstrates how having female faculty affects residents' likelihood of having children during training. Figure 4c demonstrates the non-surgical residents' perception of how their likelihood of having a child would have changed if they had chosen a surgical specialty.

Out of 110 NSS responses, 98 (89%) were between the ages of 25-34 years, and 10 (9.2%) were between 35-44 years. 92 (83.3%) identified as female, 15 (13.9%) identified as male, one (0.9%) identified as non-binary, and two (1.9%) preferred not to disclose. 93 (84.5%) did not think they had fewer opportunities to have children compared to SS. 78 (70.9%) indicated they would likely have had children later in training if they had pursued a surgical subspecialty. 62 (56.9%) female respondents indicated that having a female faculty member would impact their likelihood of having a child while in residency (Figure 4).

Surgical specialty residents' responses

**Figure 5:**
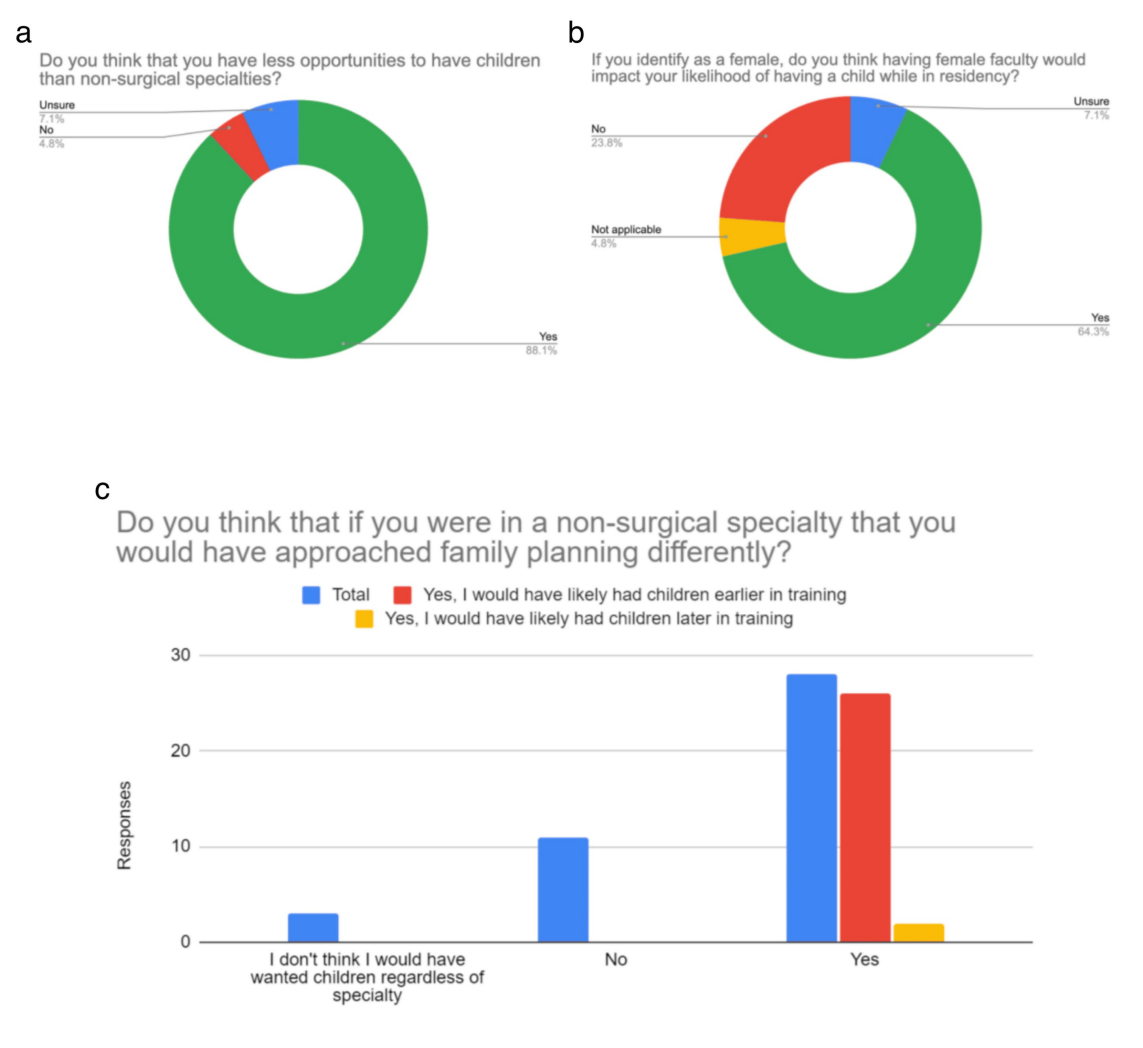
Surgical Sspecialty Residents' Responses Figure 5 shows surgical specialty residents, excluding orthopedic surgery residents. In Figure 5a, we can see the surgical residents' perceptions of having children as compared to their non-surgical counterparts. Figure 5b shows how having female faculty affects residents' likelihood of having children while in residency, and Figure 5c shows how likely surgical residents would have been to have a child during residency had they pursued a non-surgical specialty.

Out of 42 SS respondents, 39 (92.9%) were between the ages of 25 and 34 years, and three (7.1%) were between 35 and 44 years. 41 (97.6%) identified as female and one (2.4%) identified as male. 178 (88.1%) believed they had fewer opportunities to have children compared to NSS. 26 (61.9%) indicated they would likely have had children earlier in training if they had pursued an NSS. In the SS group, 28 (67.5%) female respondents indicated that having a female faculty member would impact their likelihood of having a child while in residency (Figure 5).

Orthopedic surgery residents' responses

**Figure 6:**
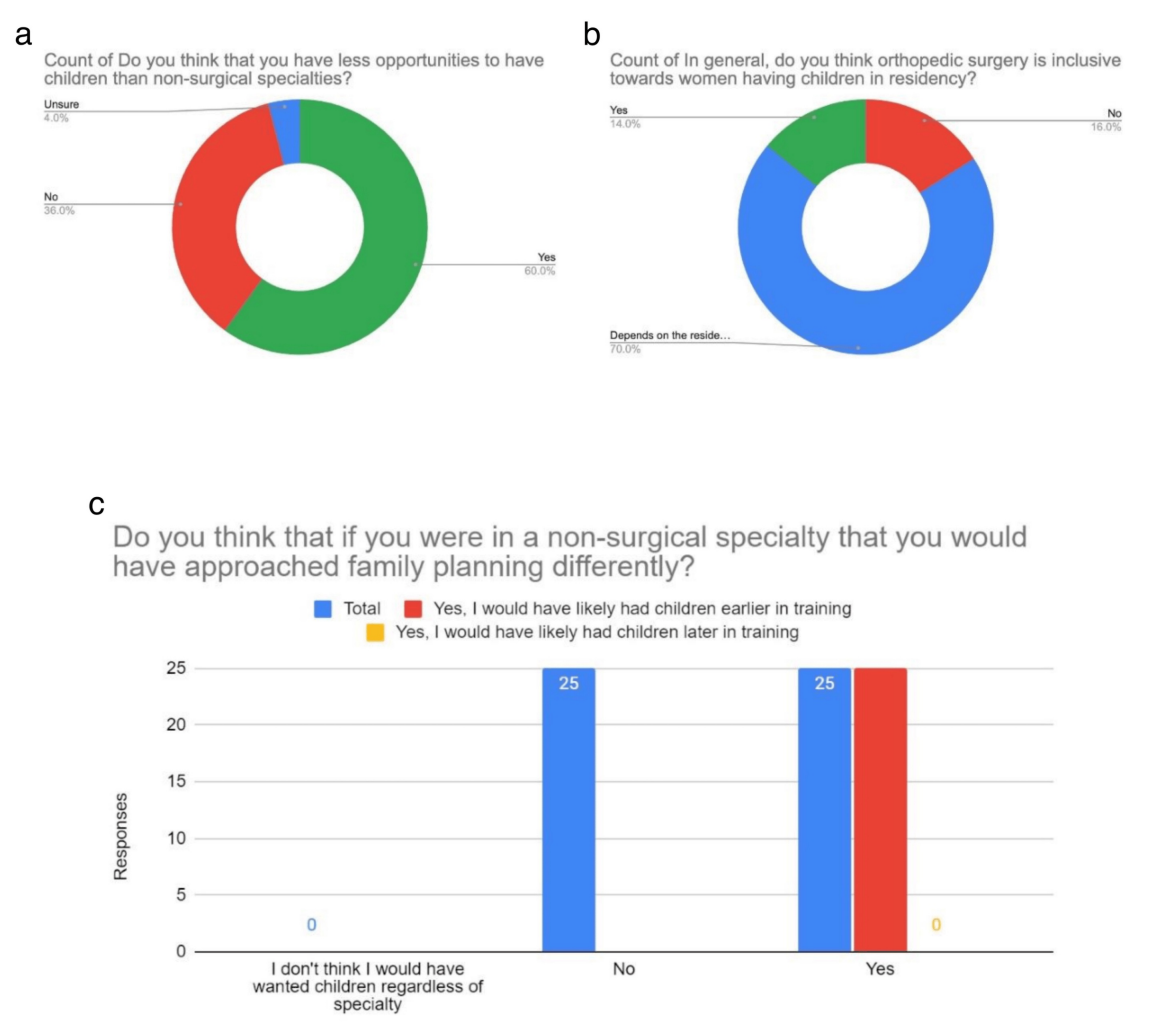
Orthopedic Surgery Residents' Responses Figure 6 shows orthopedic surgery residents' responses to our survey. In Figure 6a, we can see how many orthopedic surgery residents feel they have fewer opportunities to have children while in residency than their non-surgical counterparts. Figure 6b shows the residents' perception of how inclusive orthopedic surgery is toward women having children during residency. Figure 6c displays whether residents felt that they would have approached family planning differently had they chosen a non-surgical specialty.

Out of 50 OS resident responses, 46 (92%) were between the ages of 25 and 34, with four (8%) being between 35 and 44 years. 22 (44%) identified as female, 28 (56%) identified as male, and one (2%) identified as queer. 30 (60%) thought that they had fewer opportunities to have children compared to NSS. 25 (50%) indicated they likely would have had children earlier in training if they had pursued an NSS, while 25 (50%) indicated it would not have impacted their family planning to be in an NSS. In the OS group, 34 (68.2%) of those who identified as female indicated that having a female faculty member would impact their likelihood of having a child while in residency. On whether they thought OS was inclusive towards women having children in residency, 35 (70%) indicated that “it depends on the program,” with eight (16%) indicating “no” and seven (14%) indicating “yes” (Figure 6).

Statistics

A summary of the results is displayed in Table 1. We conducted a difference of proportions test to see if there is a statistical difference (p < 0.05) between residency programs with survey question answers. Data analysis was done using R 4.2.2 (R Foundation for Statistical Computing, Vienna, Austria). Further post hoc analysis was performed with a Bonferroni correction (Table 2).

**Table 1:** Summary Statistics A summary of the survey questions answered by respondents. Responses were grouped into one of three categories: non-surgical specialties, orthopedic surgery, and surgical specialties excluding orthopedic surgery. P-values were calculated by conducting a difference of proportions test and displayed in the table alongside the chi-squared statistic. A p-value of less than 0.05 was considered significant. *denotes statistical significance.

	Non-surgical (N=110)	Orthopedic Surgery (N=50)	Surgical (excluding Orthopedics) (N=42)	Chi-Squared Statistic	P-Value
What is your year in training?				29.74	< 0.001*
Junior	104 (94.5%)	32 (64.0%)	27 (64.3%)		
Senior	6 (5.5%)	18 (36.0%)	15 (35.7%)		
How much parental time off does your program allow that you're aware of?				8.91	0.019*
1-4 weeks	18 (16.4%)	13 (26.0%)	9 (21.4%)		
5+ weeks	73 (66.4%)	15 (30.0%)	26 (61.9%)		
Missing	19 (17.3%)	22 (44.0%)	7 (16.7%)		
Were you aware that the ACGME allows for up to 6 weeks paid time off for medical, parental, and caregiver leave prior to taking this survey?				10.46	0.005*
No	38 (34.5%)	21 (42.0%)	5 (11.9%)		
Yes	72 (65.5%)	29 (58.0%)	37 (88.1%)		
If you took parental leave, did you feel pressured to return to work earlier than anticipated?				8.18	0.225
No	26 (23.6%)	5 (10.0%)	9 (21.4%)		
Yes, by attendings	4 (3.6%)	0 (0%)	1 (2.4%)		
Yes, by co-residents	4 (3.6%)	2 (4.0%)	2 (4.8%)		
Yes, by co-residents and attendings	5 (4.5%)	6 (12.0%)	5 (11.9%)		
Missing	71 (64.5%)	37 (74.0%)	25 (59.5%)		
Do you feel supported to have a family while in residency if you chose to?				2.59	0.857
Extremely supported	31 (28.2%)	12 (24.0%)	8 (19.0%)		
Moderately supported	36 (32.7%)	18 (36.0%)	15 (35.7%)		
Neutral	19 (17.3%)	10 (20.0%)	11 (26.2%)		
Not supported	15 (13.6%)	6 (12.0%)	7 (16.7%)		
Missing	9 (8.2%)	4 (8.0%)	1 (2.4%)		
Do you feel safe to disclose family planning with your co-residents?				2.11	0.347
No	9 (8.2%)	5 (10.0%)	7 (16.7%)		
Yes	84 (76.4%)	37 (74.0%)	30 (71.4%)		
Missing	17 (15.5%)	8 (16.0%)	5 (11.9%)		
Do you feel safe to disclose family planning with your attendings/faculty?				4.31	0.115
No	23 (20.9%)	10 (20.0%)	15 (35.7%)		
Yes	58 (52.7%)	28 (56.0%)	17 (40.5%)		
Missing	29 (26.4%)	12 (24.0%)	10 (23.8%)		
Have you delayed having a family due to your specialty or medical training?				9.83	0.132
No	25 (22.7%)	21 (42.0%)	9 (21.4%)		
Yes, I delayed both in medical school and residency	44 (40.0%)	19 (38.0%)	16 (38.1%)		
Yes, I delayed while in medical school	6 (5.5%)	3 (6.0%)	2 (4.8%)		
Yes, I delayed while in residency	21 (19.1%)	5 (10.0%)	13 (31.0%)		
Missing	14 (12.7%)	2 (4.0%)	2 (4.8%)		
Have you delayed having a family due to financial barriers?				5.24	0.512
No	34 (30.9%)	26 (52.0%)	18 (42.9%)		
Yes, I delayed while in medical school	7 (6.4%)	4 (8.0%)	3 (7.1%)		
Yes, I delayed while in medical school and residency	36 (32.7%)	13 (26.0%)	13 (31.0%)		
Yes, I delayed while in residency	20 (18.2%)	6 (12.0%)	6 (14.3%)		
Missing	13 (11.8%)	1 (2.0%)	2 (4.8%)		
Did your family planning have an effect on the residency specialty you chose to pursue?				82.21	< 0.001*
No	53 (48.2%)	42 (84.0%)	30 (71.4%)		
Yes, I chose a non-surgical specialty	56 (50.9%)	0 (0%)	0 (0%)		
Yes, I chose a surgical specialty	0 (0%)	8 (16.0%)	12 (28.6%)		
Missing	1 (0.9%)	0 (0%)	0 (0%)		
Approximately how many residents are in your program?				26.37	< 0.001*
0-10	8 (7.3%)	4 (8.0%)	4 (9.5%)		
11-20	22 (20.0%)	28 (56.0%)	15 (35.7%)		
21-30	36 (32.7%)	11 (22.0%)	12 (28.6%)		
31-40	26 (23.6%)	6 (12.0%)	9 (21.4%)		
41+	18 (16.4%)	1 (2.0%)	2 (4.8%)		
Has your program ever had a resident or fellow who became pregnant during residency or fellowship?				41.56	< 0.001*
No	10 (9.1%)	21 (42.0%)	3 (7.1%)		
Yes	94 (85.5%)	18 (36.0%)	39 (92.9%)		
Missing	6 (5.5%)	11 (22.0%)	0 (0%)		
Approximately how many residents in your program have a child?				7.79	0.020*
0-5	68 (61.8%)	29 (58.0%)	35 (83.3%)		
6+	42 (38.2%)	21 (42.0%)	7 (16.7%)		
Approximately how many residents in your program identify as female?				44.92	< 0.001*
0-10	40 (36.4%)	46 (92.0%)	17 (40.5%)		
11+	70 (63.6%)	4 (8.0%)	25 (59.5%)		
Approximately how many residents in your program identify as a racial/ethnic underrepresented minority (not including as a female)? Racial/ethnic minorities include but are not limited to Hispanic/Latinos, African Americans, Asians, Native Americans, Hawaiian/Pacific Islanders				41.41	< 0.001*
0-10	58 (52.7%)	49 (98.0%)	38 (90.5%)		
11+	49 (44.5%)	1 (2.0%)	4 (9.5%)		
Missing	3 (2.7%)	0 (0%)	0 (0%)		
Does your residency program have any faculty who identify as female?				21.56	< 0.001*
No	1 (0.9%)	10 (20.0%)	2 (4.8%)		
Yes	109 (99.1%)	39 (78.0%)	40 (95.2%)		
Missing	0 (0%)	1 (2.0%)	0 (0%)		
Does your residency program have any faculty who identify as a racial/ethnic underrepresented minority (not including identifying as a female)? Racial/ethnic minorities include but are not limited to Hispanic/Latinos, African Americans, Asians, Native Americans, Hawaiian/Pacific Islanders				1.81	0.404
No	12 (10.9%)	9 (18.0%)	7 (16.7%)		
Yes	91 (82.7%)	37 (74.0%)	34 (81.0%)		
Missing	7 (6.4%)	4 (8.0%)	1 (2.4%)		
State of Residency Program				18.42	0.005*
Midwest	21 (19.1%)	16 (32.0%)	12 (28.6%)		
Northeast	18 (16.4%)	9 (18.0%)	16 (38.1%)		
Southeast	14 (12.7%)	5 (10.0%)	7 (16.7%)		
West	54 (49.1%)	20 (40.0%)	7 (16.7%)		
Missing	3 (2.7%)	0 (0%)	0 (0%)		

**Table 2:** Post Hoc Analysis Using Bonferroni’s Correction A post hoc Bonferroni correction was run to determine statistical significance between groups regarding each survey question. P-values and chi-squared statistics are displayed for each group. * denotes statistical significance. ACGME: Accrediation Council for Graduate Medical Education.

Survey Questions			Non-surgical vs. Orthopedics		Non-surgical vs. Surgical		Orthopedics vs. Surgical	
	Chi Statistic	P-value	Chi-squared Statistic	P-value	Chi-squared Statistic	P-value	Chi-squared Statistic	P-value
What is your year in training?	29.74	<0.001*	22.81	<0.001*	20.9	<0.001*	<0.001	0.999
How much parental time off does your program allow that you're aware of?	8.91	0.019*	6.57	<0.001*	0.23	0.764	2.09	0.004*
Were you aware that the ACGME allows for up to 6 weeks paid time off for medical, parental, and caregiver leave prior to taking this survey?	10.46	0.005*	0.53	0.465	6.6	0.010*	8.76	0.003*
Did your family planning have an effect on the residency specialty you chose to pursue?	82.21	<0.001*	50.3	<0.001*	55.58	<0.001*	1.44	0.229
Approximately how many residents are in your program?	26.37	<0.001*	23.92	<0.001*	6.61	0.157	4.24	0.374
Has your program ever had a resident or fellow who became pregnant during residency or fellowship?	41.56	<0.001*	30.12	<0.001*	0.02	0.270	18.97	<0.001*
Approximately how many residents in your program have a child?	7.79	0.020*	0.08	0.776	5.49	0.019*	5.77	0.016*
Approximately how many residents in your program identify as female?	44.92	<0.001*	40.59	<0.001*	0.07	0.778	25.73	<0.001*
Approximately how many residents in your program identify as a racial/ethnic underrepresented minority (not including as a female)?	41.41	<0.001*	28.12	<0.001*	15.76	<0.001*	1.26	0.261
Does your residency program have any faculty who identify as female?	21.56	<0.001*	17.1	<0.001*	0.76	0.381	3.56	0.058
State of Residency Program	18.42	0.005*	3.3	0.347	15.76	0.001*	8.49	0.036*

## Discussion

We found that residents across NSS, SS, and OS face similar barriers. Notably, 56 (27.9%) of respondents chose NSS due to family planning considerations. Key factors that increase the likelihood of pursuing a family during residency include having family nearby 154 (76.5%), being in a program where peers have had families 149 (74%), affordable childcare 139 (69%), assurance of graduation timeline with family leave 137 (68%), a program history of pregnant residents/fellows 135 (67%), and a partner as primary caregiver 106 (52.5%). 

Our survey reveals that SS, OS, and NSS residents view having a family as more challenging in surgical fields. In the NSS group, 93 (84.5%) did not feel they had fewer opportunities to have children compared to SS, with 30 (70.9%) noting they might have had children later if they had chosen a surgical subspecialty. In contrast, 37 (88.1%) of SS residents felt they had fewer opportunities to have children than NSS residents, with 26 (61.9%) suggesting they would have had children earlier if they had pursued an NSS. One respondent described switching from general surgery to emergency medicine due to family planning. Among OS residents, 25 (50%) thought they would have had children earlier if in an NSS, while the other 25 (50%) felt NSS would not have affected their family planning. Notably, OS had a higher proportion of male respondents.

When exploring the specific reasons that residents decide to delay family planning, a study in 2005 interviewed 27 family medicine residents who took maternity leave between 1994 and 1999 [10]. They found that the main reasons for the delay were long hours, unpredictable work demands, and guilt from increased workload for colleagues when taking time away from work [10]. In our survey, we found that for residents who took parental leave, 41 (20.1%) do not feel pressured to return to work, while 16 (8%) did. Our findings show improvement in residents' feeling supported to pursue family planning compared to earlier studies, but most prior studies focused on smaller subgroups and did not compare across groups [10]. 

A notable finding of our study was that financial barriers influenced 62 (30.7%) of respondents to delay starting a family. This finding could be due to the recent inflation in the economy but not the same increase in residency salary. There are limitations in the current literature to compare this specific aspect of our study to. 

Regarding the influence of gender in faculty, our survey indicates that having female faculty positively impacts the likelihood that a resident will pursue having a family while in residency. In the NSS group, 57 (56.9%) of female residents indicated that having a female faculty member would impact their likelihood of having a child while in residency (Figure 4). In the SS group, 28 (67.5%) of female residents indicated that having a female faculty member would impact their likelihood of having a child while in residency (Figure 5). In the OS group, 34 (68.2%) of female residents indicated that having a female faculty member would impact their likelihood of having a child while in residency (Figure 6). A 2013 study explored the increased birth rates among surgical residents [4]. They found that the percentage of male residents with children during residency training was similar for early and late cohorts [4]. However, for female residents, there was a substantial increase in childbearing for the late cohorts [4]. Smith et al. hypothesized that this could be due to structural changes in residency programs [4]. In our study, we found that having female faculty has a positive impact on increasing the likelihood of having a child while in residency. Having role models in the field can be of immense benefit to demonstrate how to manage family planning, especially in fields where there are limited faculty members of similar backgrounds.

Additionally, a lack of understanding regarding the availability of benefits, such as paid parental leave, may exacerbate this issue. A 2024 study found that only 10 of 170 (6%) orthopedic residency programs had policy information regarding the 6 weeks of paid time off allowed by the ACGME [11]. A recent 2025 study explored the availability of paternity leave information on program websites for US surgical residencies [12]. They found that paternity leave policies were found on only 4.3% of program-specific websites and 18.8% of Graduate Medical Education (GME) websites [12]. This study reveals that information on paternity leave is not widely available, and shows a need for standardization and transparency across all surgical specialities [12]. Our study found that 109 (54%) of respondents knew that their program allowed for six weeks of paid time off, with 48 (23.8%) being unsure of how much time off they were allowed. 31.7% of respondents in our survey were unaware that the ACGME allowed for up to 6 weeks of paid time off prior to taking the survey. Similar studies had similar findings to ours that a large majority of our respondents are unsure about time off for family planning. 

Regarding perceptions of support in family planning, 149 (74%) of respondents indicated they felt safe to disclose family planning to their co-residents, and 103 (51%) of respondents indicated they felt safe to disclose family planning with their faculty (Figure 2), demonstrating the importance of professional barriers in discussing family planning. A SS resident wrote, “Regardless of what your co-residents say and how supported they try to make you feel, there is still pressure to come back from maternity leave and be part of the call pool.” In a 2023 systematic review, they noted “negative perceptions of pregnancy from fellow trainees” and program directors as likely contributors to voluntarily delaying childbearing [8]. Regarding the respondent’s perception of support to have a family in residency, 69 (34.3%) indicated “moderately supported,” 51 (25.4%) indicated “extremely supported,” 40 (19.9%) indicated “neutral,” and 28 (13.9%) indicated “not supported” (Figure 2). A 2024 study reported that female surgeon infertility rates are approximately 32% compared to 10.9% in the general population, which is likely secondary to voluntary delaying childbearing and occupational stressors [13]. A SS resident wrote, “Pregnant women are seen as less hard working, more likely to have adverse pregnancy outcomes, and are not supported in the postpartum period.” Another study reported that (65%) of female surgeons delay childbearing [13].

Whether the OS resident group in our survey thought OS was inclusive towards women having children during residency, 35 (70%) indicated that “it depends on the program,” with eight (16%) indicating “no” and seven (14%) indicating “yes” (Figure 6). This highlights the impact of program selection, faculty, and culture of a program on an individual’s decisions regarding family planning. The challenges of family planning in residency, as indicated in our survey responses and prior literature, demonstrate that being a physician causes barriers to family planning across specialties, especially in surgical subspecialties like orthopedic surgery.

Strengths and limitations of the study

This study offers strengths in understanding family planning during residency, with its nationwide survey encompassing residents from a diverse range of specialties and postgraduate years, enhancing generalizability. By including non-surgical specialties and surgical specialties, the study provides a comprehensive overview and a targeted focus on comparison with orthopedic surgery, a historically male-dominated field. Utilizing Instagram and email for distribution broadened the participant pool, promoting inclusivity, though this method of distribution may have limited our pool of respondents to those who are active on Instagram. Additionally, the anonymous survey format encouraged candid responses on sensitive topics, reducing social desirability bias. The integration of qualitative and quantitative questions allowed for a nuanced exploration of residents’ experiences. Notably, the study examines gender and specialty-specific issues, particularly in orthopedic surgery, where female representation has historically been low. This study addresses a research gap to enhance understanding of how different residency fields impact family planning decisions. However, limitations include potential response bias, with participants possibly influenced by social desirability or recall bias. The use of social media for recruitment may also skew results toward more active online residents. A large proportion of respondents were located on the west coast of the United States, which may be a source of regional bias. The small sample size, along with an uneven distribution favoring non-surgical specialties, limits the ability to detect subtle differences and may affect comparability across specialties. Additionally, recall bias and the survey’s snapshot nature limit insights into long-term outcomes and specific barriers faced by different specialties. Institutional policy and cultural differences further constrain the generalizability of findings across all U.S. residency programs.

Future research directions

Future research should explore family planning experiences across a broader range of medical specialties, particularly smaller or less studied fields like orthopedic and neurological surgery, which historically have been male-dominated fields. This study highlights the need to identify specialty-specific barriers affecting residents’ family planning decisions. Longitudinal studies could track how perceptions and decisions evolve over training, including attitudes towards parental leave, institutional support, and work-life balance. Investigating the impact of supportive leadership and institutional changes on family planning within various specialties could provide deeper insights. Research should also address the intersection of family planning with gender, marital status, and socioeconomic factors affecting residents’ ability to balance work and family. Examining the roles of mentorship in family planning could lead to actionable improvements in residency programs. The study suggests the need for multi-institutional or national research to validate trends and provide a comprehensive understanding across different settings. Standardizing policies and increasing transparency in parental leave and support options could create more equitable experiences for residents. Lastly, targeted interventions like policy reforms, improved child care resources, and leadership changes should be explored to alleviate pressures and foster supportive environments for residents navigating family planning during their training. Future research studies should examine how policies affect family planning over time, as opposed to this cross-sectional study. 

## Conclusions

Addressing family planning challenges in residency requires a thorough examination as residents balance demanding medical training with personal life decisions. This survey of 202 residents nationwide reveals notable disparities in support for family planning across specialties, with both surgical and non-surgical specialties perceiving family planning as more challenging in surgical fields like orthopedic surgery. It also shows that having a female faculty is positively correlated with female residents’ decisions to start a family during residency. These findings underscore the need for equitable, gender-sensitive policies and flexible parental leave options to support residents, especially women in male-dominated fields. Implementing comprehensive resources for work-life balance and conducting longitudinal and multi-institutional studies will be crucial for validating these findings and improving support across diverse training environments. Addressing these issues is vital for enhancing resident well-being and fostering a more inclusive medical training environment.

## Figures and Tables

**Figure 7:**
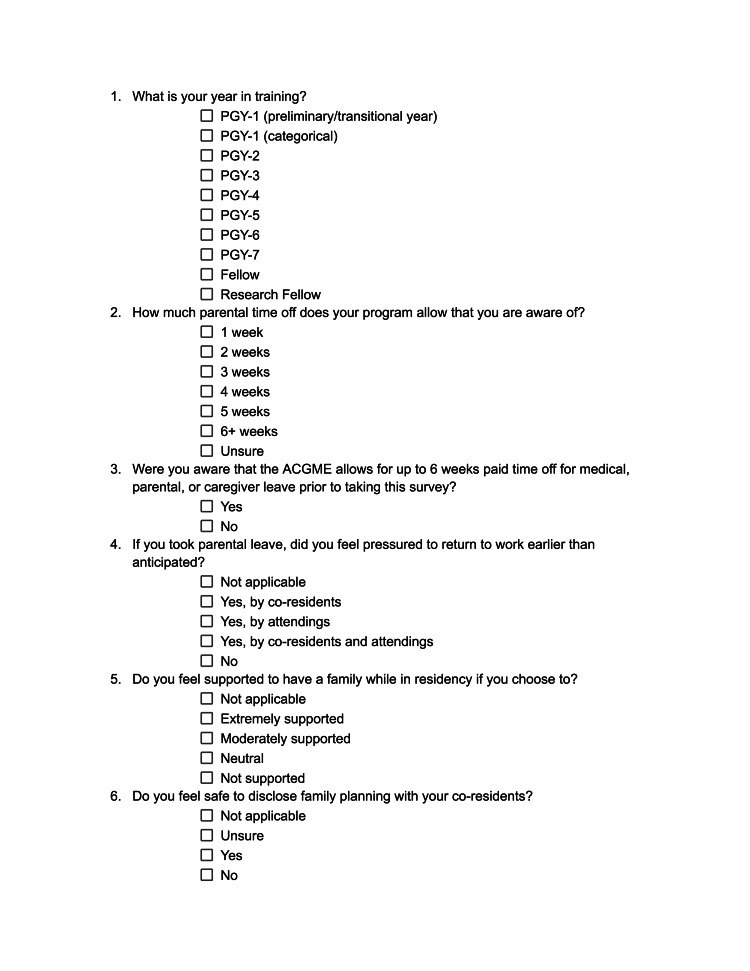
Survey Page 1

**Figure 8:**
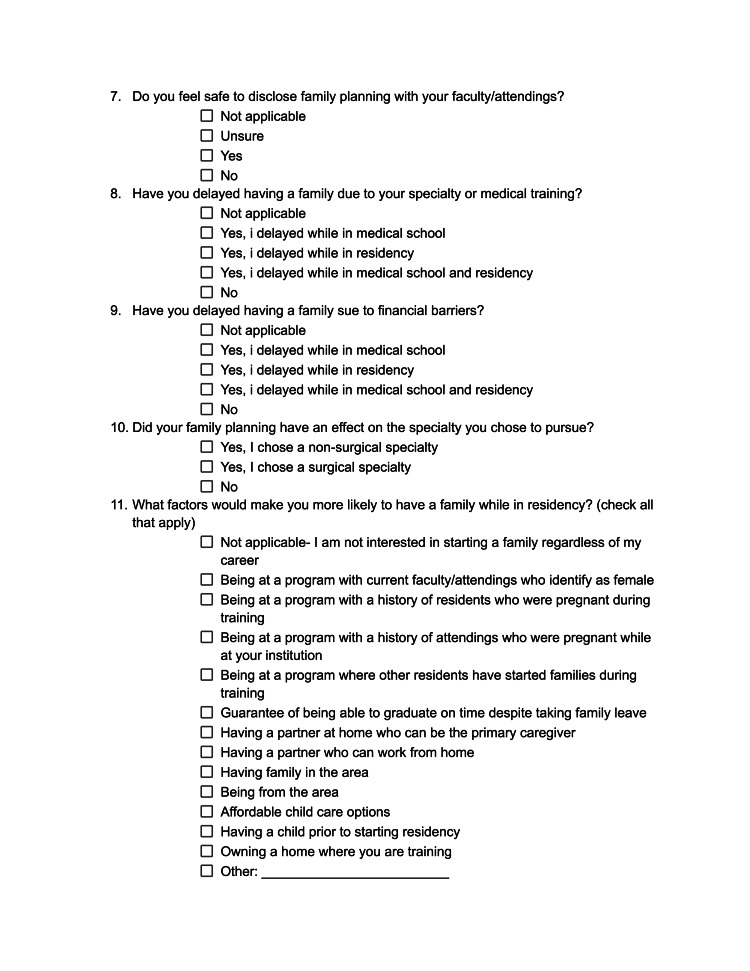
Survey Page 2

**Figure 9:**
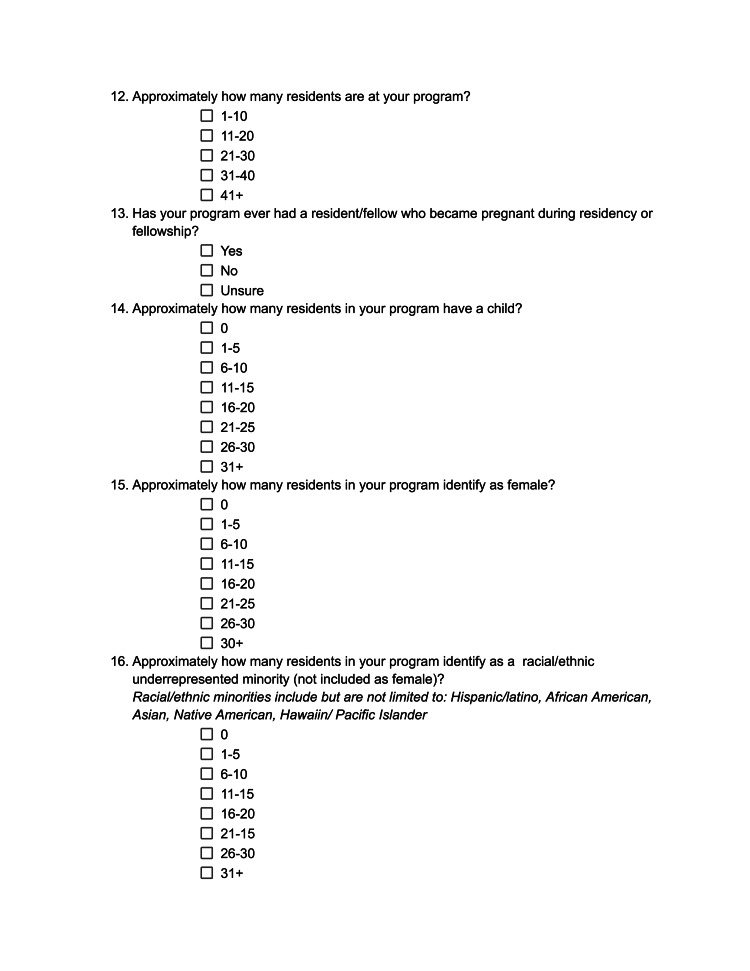
Survey Page 3

**Figure 10:**
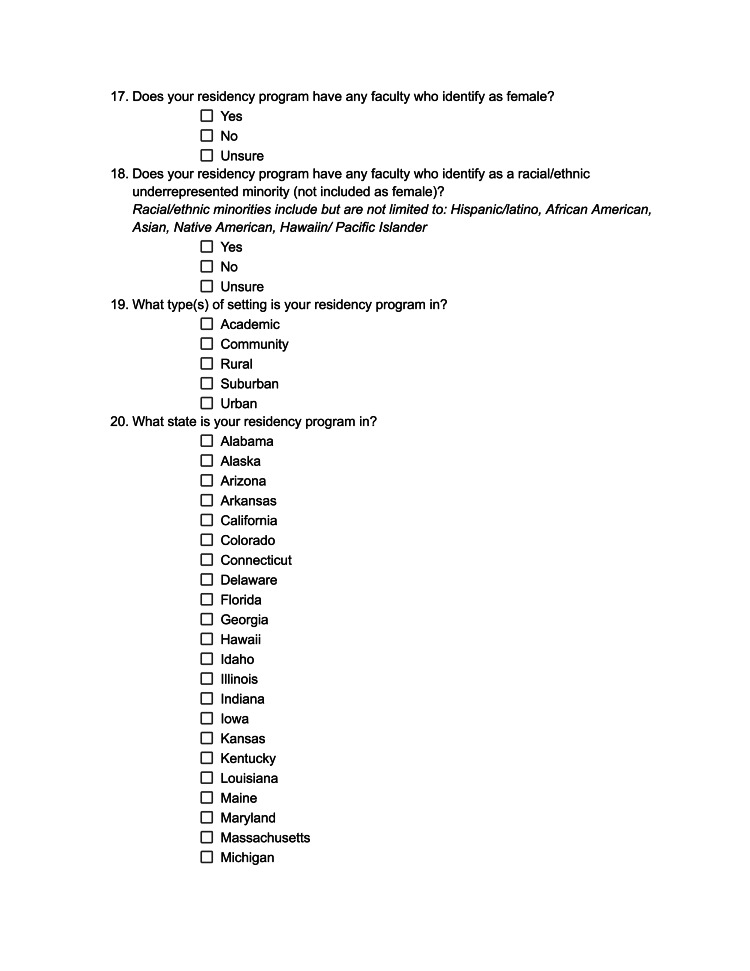
Survey Page 4

**Figure 11:**
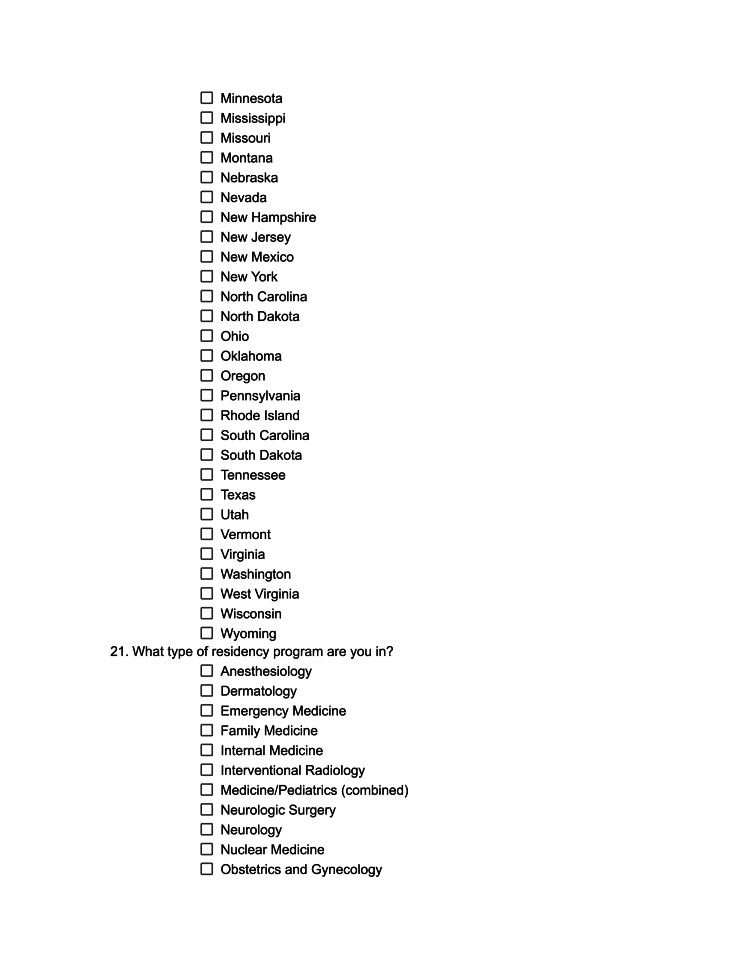
Survey Page 5

**Figure 12:**
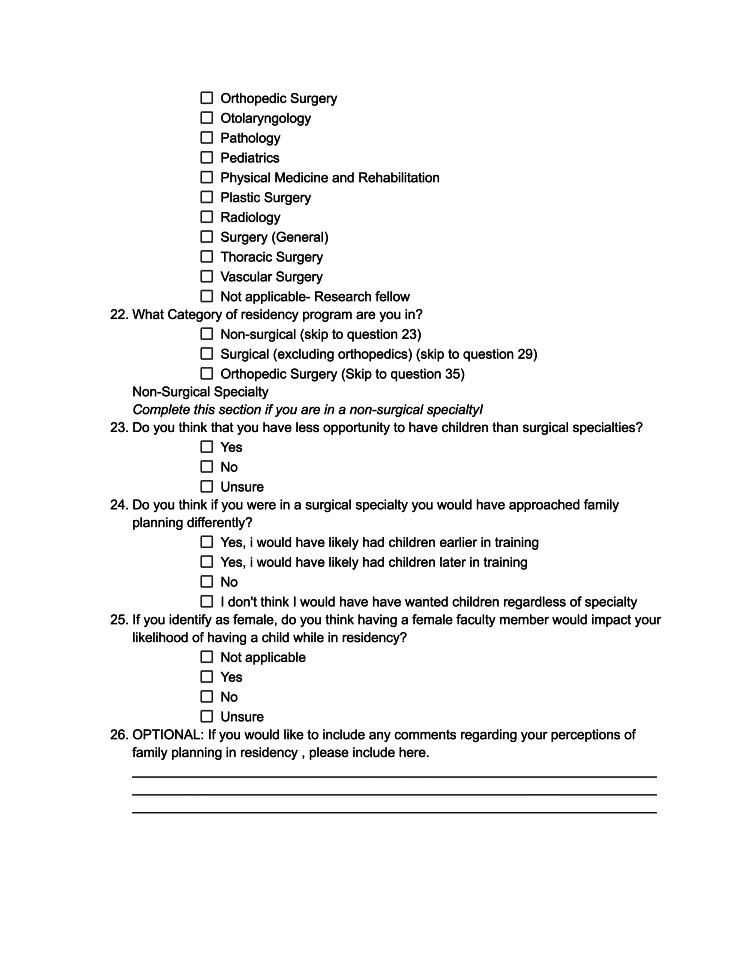
Survey Page 6

**Figure 13:**
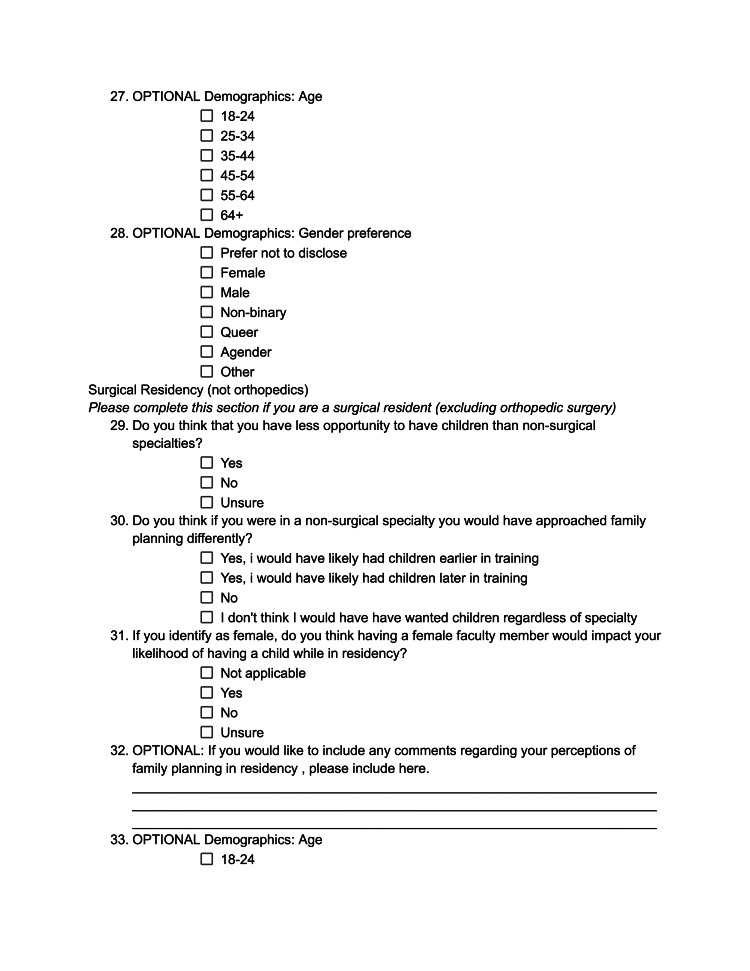
Survey Page 7

**Figure 14:**
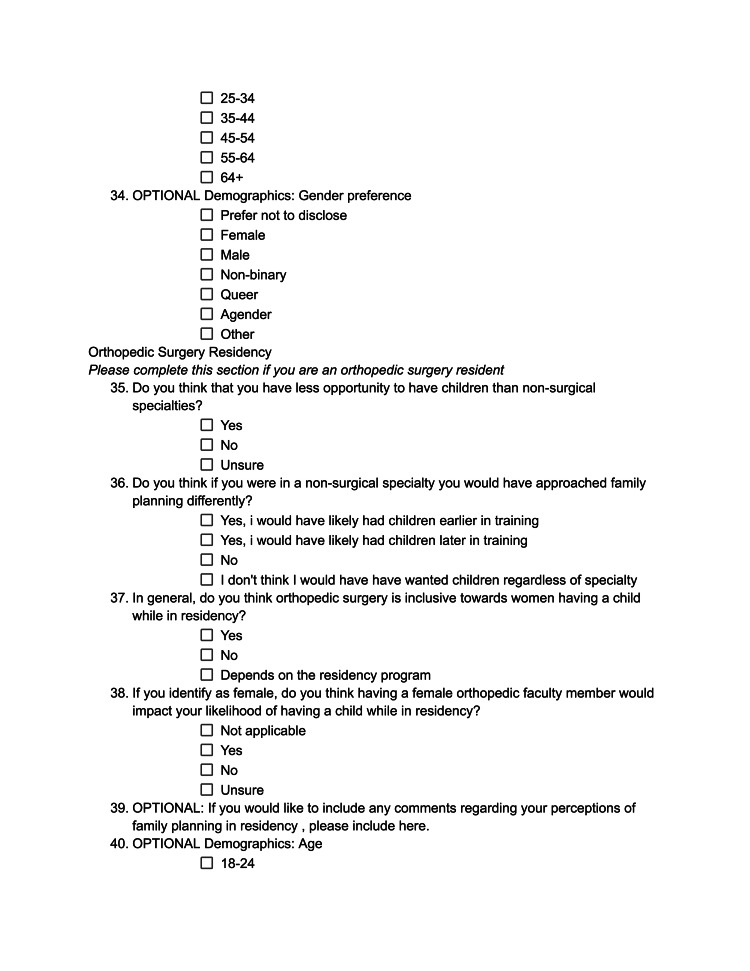
Survey Page 8

**Figure 15:**
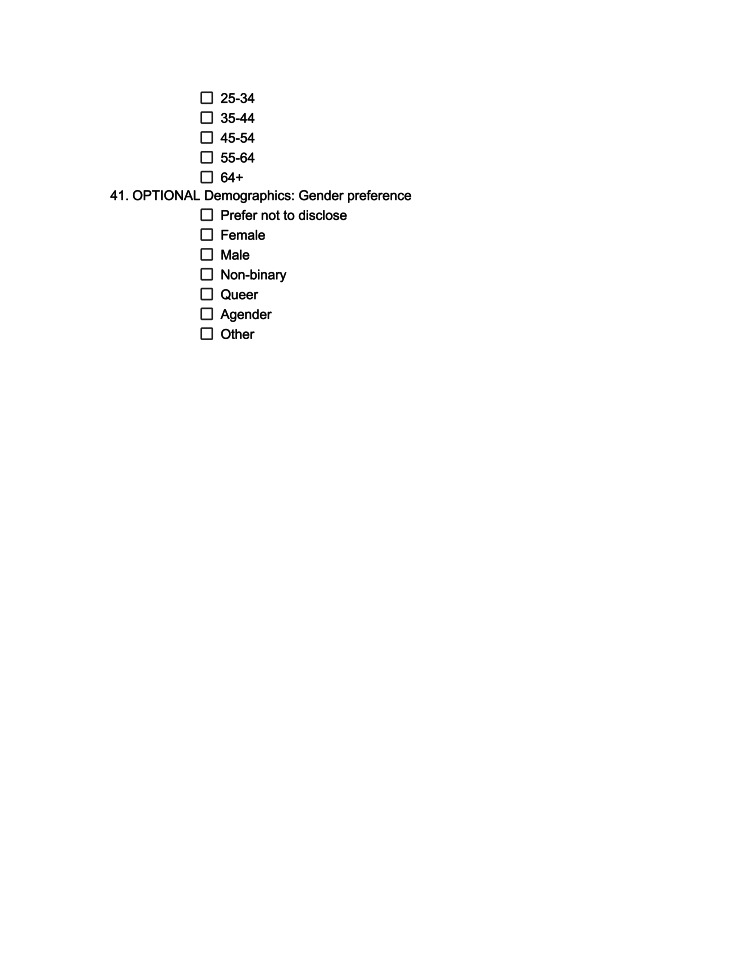
Survey Page 9

## Appendices

 Credits: Janae Rasmussen, DO and Noah Paisner, DO original creation
